# Supplementary material for: Post-marketing safety of solriamfetol: A retrospective pharmacovigilance study based on the us food and drug administration adverse event reporting system
Source: PLoS One. 2025 Sep 22;20(9):e0333130. doi: 10.1371/journal.pone.0333130 (PMC12453233; doi:10.1371/journal.pone.0333130)
Supplement: S1 Table — (DOCX) [file pone.0333130.s001.docx]

**S1 Table. Four major algorithms used for signal detection.**

| Algorithms | Equation | Criteria |
| --- | --- | --- |
| ROR | ROR=(a/c)/(b/d)=ad/bc | lower limit of 95% CI>1, N≥3 |
|  | 95%CI=e^ln(ROR)±1.96(1/a+1/b+1/c+1/d)^0.5^ |  |
| PRR | PRR=[a/(a+b)]/[c/(c+d)] | PRR≥2, χ^2^≥4, N≥3 |
|  | χ^2^=[(ad-bc)^2](a+b+c+d)/[(a+b)(c+d)(a+c)(b+d)] |  |
| BCPNN | IC=log_2_(a(a+b+c+d)/(a+b)/(a+c)) | IC_025_>0 |
|  | IC_025_=e^ln(IC)−1.96(1/a+1/b+1/c+1/d)^0.5^ |  |
| MGPS | EBGM=a(a+b+c+d)/ [(a+c)(a+b)] | EBGM05>2, N>0 |
|  | 95%CI=e^ln(EBGM)±1.96(1/a+1/b+1/c+1/d)^0.5^ |  |

Equation: a) Number of reports containing both the target drug and target adverse drug reaction. b) Number of reports on the other adverse drug reactions of the target drug. c) Number of reports on the target adverse drug reaction of other drugs. d) Number of reports containing other drugs and adverse drug reactions. CI, confidence interval; *N*, number of reports; χ^2^, chi-square test; IC, information component; IC_025_, the lower limit of 95% CI of the IC; EBGM, empirical Bayesian geometric mean; EBGM05, lower limit of the 95% CI of empirical Bayesian geometric mean.
