# Supplementary material for: Post-marketing safety of solriamfetol: A retrospective pharmacovigilance study based on the us food and drug administration adverse event reporting system
Source: PLoS One. 2025 Sep 22;20(9):e0333130. doi: 10.1371/journal.pone.0333130 (PMC12453233; doi:10.1371/journal.pone.0333130)
Supplement: S2 Table — (DOCX) [file pone.0333130.s002.docx]

**S2 Table. Signal Strength of Adverse Events Associated with Solriamfetol for the Treatment of Narcolepsy: Ranked by Number of Reports at the PT Level in the FAERS Database.**

| Preferred Terms | System Organ Class | N | ROR(95% CI) | PRR(95% CI) | χ2 | IC(IC025) | EBGM(EBGM05) |
| --- | --- | --- | --- | --- | --- | --- | --- |
| Drug ineffective | General disorders and administration site conditions | 149 | 11.37(9.58, 13.5) | 10.24(8.75, 11.98) | 1235.75 | 3.34(3.09) | 10.09(8.74) |
| Headache | Nervous system disorders | 54 | 4.16(3.17, 5.46) | 4.03(3.12, 5.2) | 123.65 | 2.01(1.62) | 4.01(3.19) |
| Anxiety | Psychiatric disorders | 47 | 4.16(3.11, 5.57) | 4.05(3.08, 5.33) | 108.34 | 2.01(1.6) | 4.03(3.16) |
| Palpitations | Cardiac disorders | 19 | 6.26(3.97, 9.86) | 6.18(3.94, 9.7) | 81.92 | 2.62(1.98) | 6.13(4.19) |
| Exposure during pregnancy | Injury, poisoning and procedural complications | 17 | 26.69(16.38, 43.5) | 26.37(16.15, 43.04) | 398 | 4.66(3.98) | 25.32(16.83) |
| Blood pressure increased | Investigations | 17 | 4.72(2.92, 7.62) | 4.67(2.92, 7.47) | 48.79 | 2.21(1.54) | 4.64(3.11) |
| Therapeutic response decreased | General disorders and administration site conditions | 13 | 15.48(8.9, 26.9) | 15.34(8.86, 26.56) | 170.1 | 3.91(3.14) | 14.99(9.44) |
| Therapeutic response shortened | General disorders and administration site conditions | 13 | 16.63(9.56, 28.93) | 16.48(9.52, 28.53) | 184.23 | 4.01(3.24) | 16.08(10.12) |
| Feeling jittery | General disorders and administration site conditions | 12 | 34.89(19.46, 62.57) | 34.6(19.22, 62.29) | 370.63 | 5.04(4.23) | 32.8(20.12) |
| Migraine | Nervous system disorders | 12 | 3.6(2.04, 6.37) | 3.58(2.03, 6.32) | 22.21 | 1.83(1.04) | 3.56(2.21) |
| Agitation | Psychiatric disorders | 11 | 5.72(3.15, 10.38) | 5.68(3.15, 10.23) | 42.12 | 2.5(1.67) | 5.64(3.43) |
| Therapeutic response unexpected | General disorders and administration site conditions | 11 | 21.24(11.62, 38.83) | 21.08(11.48, 38.7) | 203.43 | 4.35(3.52) | 20.41(12.32) |
| Tachycardia | Cardiac disorders | 11 | 6.2(3.42, 11.26) | 6.16(3.42, 11.09) | 47.16 | 2.61(1.79) | 6.11(3.71) |
| Heart rate increased | Investigations | 11 | 3.68(2.03, 6.67) | 3.66(2.03, 6.59) | 21.14 | 1.86(1.04) | 3.64(2.21) |
| Suicidal ideation | Psychiatric disorders | 11 | 4.54(2.5, 8.24) | 4.52(2.51, 8.14) | 29.94 | 2.17(1.34) | 4.49(2.73) |
| Irritability | Psychiatric disorders | 10 | 5.04(2.7, 9.41) | 5.01(2.68, 9.38) | 31.88 | 2.32(1.46) | 4.98(2.95) |
| Wrong technique in product usage process | Injury, poisoning and procedural complications | 8 | 6.32(3.15, 12.72) | 6.29(3.17, 12.49) | 35.29 | 2.64(1.69) | 6.24(3.48) |
| Product administration interrupted | Injury, poisoning and procedural complications | 7 | 16.04(7.56, 34.04) | 15.96(7.58, 33.61) | 95.72 | 3.96(2.94) | 15.58(8.3) |
| Pre-existing condition improved | General disorders and administration site conditions | 5 | 22.38(9.16, 54.73) | 22.31(9.24, 53.89) | 98.19 | 4.43(3.25) | 21.56(10.2) |
| Abortion spontaneous | Pregnancy, puerperium and perinatal conditions | 5 | 102.24(39.61, 263.9) | 101.87(39.76, 260.99) | 428.07 | 6.45(5.2) | 87.46(39.56) |
| Prescribed overdose | Injury, poisoning and procedural complications | 5 | 6.47(2.68, 15.63) | 6.45(2.67, 15.58) | 22.79 | 2.68(1.51) | 6.39(3.05) |
| Nephrolithiasis | Renal and urinary disorders | 5 | 7.7(3.18, 18.64) | 7.68(3.18, 18.55) | 28.7 | 2.93(1.76) | 7.6(3.63) |
| Cataplexy | Nervous system disorders | 5 | 9.15(3.78, 22.17) | 9.12(3.78, 22.03) | 35.64 | 3.17(2) | 9(4.29) |
| Drug dose titration not performed | Injury, poisoning and procedural complications | 3 | 306.27(76.52, 1225.89) | 305.6(76, 1228.9) | 607.22 | 7.67(6) | 204.07(63.94) |
| Mania | Psychiatric disorders | 3 | 7.2(2.31, 22.51) | 7.19(2.31, 22.41) | 15.81 | 2.83(1.4) | 7.12(2.74) |
| Uterine leiomyoma | Neoplasms benign, malignant and unspecified | 3 | 32.24(10.08, 103.06) | 32.17(10.12, 102.25) | 86.08 | 4.94(3.47) | 30.61(11.58) |
| Sleep attacks | Psychiatric disorders | 3 | 23.56(7.43, 74.73) | 23.51(7.4, 74.73) | 62.26 | 4.5(3.05) | 22.67(8.63) |
| Persecutory delusion | Psychiatric disorders | 3 | 32.81(10.26, 104.96) | 32.74(10.3, 104.06) | 87.63 | 4.96(3.5) | 31.13(11.77) |

Abbreviations: ROR, Reporting Odds Ratio; PRR, Proportional Reporting Ratio; IC, Information Component; EBGM, Empirical Bayes Geometric Mean.
