# Supplementary material for: Post-marketing safety of solriamfetol: A retrospective pharmacovigilance study based on the us food and drug administration adverse event reporting system
Source: PLoS One. 2025 Sep 22;20(9):e0333130. doi: 10.1371/journal.pone.0333130 (PMC12453233; doi:10.1371/journal.pone.0333130)
Supplement: S3 Table — (DOCX) [file pone.0333130.s003.docx]

**S3 Table. Signal Strength of Adverse Events Associated with Solriamfetol for the Treatment of Obstructive Sleep Apnea: Ranked by Number of Reports at the PT Level in the FAERS Database**

| Preferred Terms | System Organ Class | N | ROR(95% CI) | PRR(95% CI) | χ2 | IC(IC025) | EBGM(EBGM05) |
| --- | --- | --- | --- | --- | --- | --- | --- |
| Drug ineffective | General disorders and administration site conditions | 14 | 5.45(3.19, 9.33) | 5.23(3.14, 8.71) | 48.36 | 2.39(1.64) | 5.23(3.34) |
| Headache | Nervous system disorders | 11 | 4.85(2.65, 8.86) | 4.7(2.61, 8.46) | 32.27 | 2.23(1.4) | 4.7(2.84) |
| Anxiety | Psychiatric disorders | 9 | 6.72(3.46, 13.05) | 6.53(3.42, 12.47) | 42.35 | 2.71(1.8) | 6.53(3.75) |
| Disturbance in attention | Nervous system disorders | 4 | 11.4(4.25, 30.62) | 11.26(4.23, 30) | 37.37 | 3.49(2.21) | 11.24(4.92) |
| Feeling abnormal | General disorders and administration site conditions | 4 | 4.64(1.73, 12.45) | 4.59(1.72, 12.23) | 11.25 | 2.2(0.92) | 4.59(2.01) |
| Somnolence | Nervous system disorders | 4 | 5.06(1.89, 13.59) | 5.01(1.88, 13.35) | 12.85 | 2.32(1.05) | 5(2.19) |
| Migraine | Nervous system disorders | 4 | 8.92(3.32, 23.95) | 8.81(3.31, 23.47) | 27.7 | 3.14(1.86) | 8.8(3.85) |
| Therapeutic response decreased | General disorders and administration site conditions | 3 | 20.46(6.55, 63.94) | 20.26(6.5, 63.15) | 54.79 | 4.34(2.91) | 20.2(7.79) |
| Therapeutic response unexpected | General disorders and administration site conditions | 3 | 43.56(13.91, 136.34) | 43.11(13.83, 134.36) | 122.66 | 5.42(3.99) | 42.85(16.49) |
| Narcolepsy | Nervous system disorders | 3 | 234.76(73.87, 746.07) | 232.29(74.53, 724) | 668.64 | 7.81(6.36) | 224.83(85.45) |
| Feeling jittery | General disorders and administration site conditions | 3 | 60.88(19.42, 190.84) | 60.25(19.33, 187.79) | 173.33 | 5.9(4.47) | 59.74(22.97) |
| Palpitations | Cardiac disorders | 3 | 6.95(2.23, 21.7) | 6.89(2.25, 21.06) | 15.12 | 2.78(1.36) | 6.88(2.66) |
| Sleep attacks | Psychiatric disorders | 3 | 270.88(85, 863.3) | 268.03(84.33, 851.93) | 768.58 | 8.01(6.55) | 258.14(97.87) |

Abbreviations: ROR, Reporting Odds Ratio; PRR, Proportional Reporting Ratio; IC, Information Component; EBGM, Empirical Bayes Geometric Mean.
