# Supplementary material for: Post-marketing safety of solriamfetol: A retrospective pharmacovigilance study based on the us food and drug administration adverse event reporting system
Source: PLoS One. 2025 Sep 22;20(9):e0333130. doi: 10.1371/journal.pone.0333130 (PMC12453233; doi:10.1371/journal.pone.0333130)
Supplement: Attachment — Submitted filename: Response to Reviewers.docx [file pone.0333130.s005.docx]

**27-May-2025**

**Dear Prof. Christian Veauthier,**

Thank you for your time and valuable feedback on our manuscript entitled **"Post-marketing Safety of Solriamfetol: A Retrospective Pharmacovigilance Study Based on the US Food and Drug Administration Adverse Event Reporting System" (Submission ID PONE-D-25-14184)**. We have carefully addressed all comments and suggestions. Modifications in the revised manuscript are highlighted in red, and point-by-point responses are provided below:

**Responds to the reviewer’s comments:**

**Reviewer: 1**

**Comment 1**

The study analizes AEs of solriamfetol retrospectively using real-world data.

P4 line 55: Authors should clearly state if they refer to EDS in general, in narcolepsy or in OSA. Otherwise the mentioning of specific wake promoting agents does not make sense, since they are approved for certain indications only. Pitolisant as alternative has not been mentioned.

**Response: *Thank you for this valuable comment. We agree that it is essential to clarify the indication when discussing wake-promoting agents. In the revised manuscript, we have clearly specified that. Additionally, we have added pitolisant as another approved treatment for EDS in narcolepsy. (P4 line 58-63)***

**Comment 2**

Table 1: it should be mentioned that the prescription in Europe is much more restricted to the approved indications as in the US, which could attribute to the high amount of AEs in the US.

**Response:** ***Thank you for this insightful comment. We agree that differences in regulatory approvals and prescribing practices between the United States and Europe could have influenced the geographic distribution of adverse event reports. Accordingly, we have added a sentence in the discussion section highlighting that solriamfetol prescriptions are more restricted to approved indications in Europe, which may partly explain the predominance of reports from the United States. (P22 line 206-210)***

**Comment 3**

Table 2 needs a legend of abbreviations. The AEs are rather unspecfic. There seems to be much more information about different psychiatric disorders and cardiac symptoms as can be seen in table 3. Could drug ineffectiveness be dependant on dosage or comedication? There is no information about this. Therefore it would be nice to have some more information about the settings of the FAERS database.

**Response: *Thank you for these important and constructive suggestions. We have taken the following actions:***

***(1) A legend of abbreviations (ROR, PRR, IC, EBGM) has been added to the footnote of Table 2 and Table 3 for clarity.***

***(2) We agree that more detailed adverse events are described in Table 3 using Preferred Terms (PTs), which provides greater specificity, particularly for psychiatric and cardiac-related symptoms.***

***(3) Regarding drug ineffectiveness, we acknowledge that factors such as dosage, comedication, and adherence could influence perceived treatment outcomes. However, due to the limitations of the FAERS database, specific data on dose and concomitant medications are often missing or incomplete. We have added a note in the discussion section to reflect this limitation. (P23 line 242-247)***

**Comment 4**

What are general disorders, please explain in more detail.

**Response: *Thank you for pointing this out. We agree that “general disorders and administration site conditions” is a broad MedDRA SOC term that may require clarification. In our study, adverse events falling under this category primarily included drug ineffectiveness, decreased therapeutic response, feeling jittery, and unexpected therapeutic response. These terms reflect systemic reactions or general treatment dissatisfaction, rather than organ-specific symptoms. We have added this explanation to the manuscript for better clarity.*** ***(P23 line 226-229)***

**Comment 5**

What do you consider to be poisoning? Is this term directly related to intake of solriamfetol only or is it including all preexisting forms of medication, alcohol and drug abuse etc.?

**Response: *Thank you for raising this important question. In our analysis, the term “poisoning” falls under the broader MedDRA System Organ Class “Injury, poisoning and procedural complications,” which includes a wide range of adverse events. In the FAERS database, this classification may refer to cases of overdose, drug misuse, medication errors, or intoxication, and not necessarily caused by solriamfetol alone. However, in our study, this SOC contained only 2 of the top 30 PTs, namely Exposure during pregnancy and Interrupted product administration.***

**Comment 6**

I suggest to give table 3 a better structure by listing all psychiatric disorders first according to frequency followed by nervous system disorders etc.

**Response: *Thank you for this very helpful suggestion. We have revised Table 3 accordingly. (P15 – P20)***

**Comment 7**

P19 line 163: Since most AEs were reported within the first 7 days it would be good to discuss if these were in fact substance related or unspecific. Moreover the most important AE for women would be spontaneous abortion. This needs special consideration, because it would imply not to prescribe solriamfetol for women with child bearing potential.

**Response: *Thank you for this important comment. We agree that the high proportion of adverse events (AEs) occurring within the first 7 days of solriamfetol use warrants careful interpretation. We have now addressed this in the discussion section, noting that early-onset AEs may be substance-related due to initial pharmacodynamic effects, but non-specific reactions such as nocebo effects or anxiety at treatment initiation cannot be excluded. (P23 line 237-242) Additionally, we appreciate your point regarding spontaneous abortion. We have expanded the discussion to emphasize this serious AE, especially in women of childbearing potential. Given that solriamfetol is not recommended during pregnancy and safety data are limited, prescribing it to women of reproductive age should involve a thorough risk–benefit assessment and consideration of pregnancy prevention measures. (P26 line 288-294)***

**Comment 8**

Given all the limitation of the FAERS database at the end of the discussion, the conclusion should be much more cautious. It should mention that the most common side effets of the initial solriamfetol studies were confirmed. I suggest to mention the study limitations clearly in the method chapter to shed a light on the presented results, instead of presenting them at the very end.

**Response: *Thank you for your insightful suggestions. We fully agree that the limitations of the FAERS database warrant a more cautious interpretation of the findings. Accordingly, we have revised the conclusion to present a more conservative tone, emphasizing that while the study confirms several known adverse events from earlier clinical trials, causality cannot be established due to the nature of spontaneous reporting data. We also moved a brief summary of the database limitations to the Methods section to help contextualize the findings for readers from the outset. We appreciate this suggestion, which improves the overall transparency and scientific rigor of the manuscript. (P6 line 95-99)***

**Comment 9**

The discussion should certainly comment the decline of AEs from 2023 on.

**Response: *Thank you for this important observation. We agree that the decline in reported adverse events (AEs) related to solriamfetol from 2023 onwards warrants discussion. We have now added a paragraph in the discussion section addressing this trend. Possible explanations include a stabilization in prescribing practices, better physician-patient education, underreporting over time, or a natural reporting attenuation as the drug becomes more established in clinical practice. Due to the nature of the FAERS database, however, we cannot determine the precise cause of this decline. (P22 line 211-218)***

**Reviewer: 2**

**Comment 1**

Funding: Please elaborate further on the funding of this project. I could not find any additional information on the Grant No. H202311.

**Response: *Thank you for your comment regarding the funding information. We apologize for the lack of detail. The study was supported by a research grant from the Jiangsu Pharmaceutical Association under Grant No. H202311 (Details are available at http://www.jspa.org.cn/xwzx/tzgg/202311/t20231128_1415401.html)***

**Comment 2**

Abstract: “EDS…and may be life-threatening”. This is true in the context of car accident etc., but the wording here is a little unfortunate. Please rephrase this sentence.

**Response: *Thank you for your helpful observation. We agree that the original phrasing may be interpreted as overly broad. We have revised the sentence to more accurately reflect that excessive daytime sleepiness (EDS) may increase the risk of life-threatening situations, particularly in contexts such as driving or operating heavy machinery. (P2 line 14-15)***

**Comment 3**

Introduction: For a better description of what is EDS you should consider Lammers et al., Sleep Med Rev, 2020.

**Response: *Thank you for this valuable suggestion. We agree that the review by Lammers et al. provides an authoritative and comprehensive overview of excessive daytime sleepiness (EDS). We have now cited this reference in the Introduction section to support the definition and conceptual framework of EDS. (P4 line 40-43)***

**Comment 4**

Introduction: I disagree with your statement “Although continuous positive airway pressure is effective, EDS may persist in patients with OSA, almost all of whom undergo pharmacological therapy [8]”. Please provide data on this statement. I´m not aware of such data. Only some patients undergo pharmacological therapy, not “almost all”

**Response: *Thank you for pointing this out. We agree that the statement was too strong and have revised it accordingly. While persistent EDS in patients with OSA is recognized in clinical practice, only a subset of patients undergo pharmacological treatment. The text has been corrected to reflect this. (P4 line 50-57)***

**Comment 5**

Introduction: Next, you describe “Modafinil and armodafinil promote arousal and are currently used as first-line treatments for EDS in adults”. The reference you provide does not refer to EDS in OSA or in narcolepsy. You´re not precise here and are mixing up different things. Please clarify in the introduction what are current pharmacological recommendations for EDS in narcolepsy, for EDS in OSA and for other, if available.

**Response: *We appreciate this comment and agree that the initial description lacked precision. We have revised the Introduction to clearly distinguish between pharmacologic recommendations for EDS in narcolepsy and OSA. Updated references have been included to support these distinctions. (P4 line 58-63)***

**Comment 6**

Methods/ Discussion/ Conclusions: Limitation of FEARS database: It includes data from “various sources, including medical professionals, consumers,…”. And as described in the results, mostly consumers (>50%) reported AEs. The FEARS database itself describes all its limitations on their webpage. You mention most, but not all of them. Further, for a lot of data “unknown” or “other” is described. I.e. you cannot make strong statement on age “median age was 40”, if 78% of data on age are “unknown”. This also refers to other points in your discussion.

**Response: *Thank you for highlighting this. We have updated the Discussion sections to include additional limitations as described by the FAERS database, including high proportions of missing data and the predominance of consumer-submitted reports. We have also revised the statements regarding median age and other variables with substantial missing data to avoid overinterpretation. (P22 line 199-203)***

**Comment 7**

The discussion is too long and repeats results.

**Response: *Thank you for this suggestion. We have shortened the Discussion section by removing repetitive descriptions of the results and focusing more on the interpretation, implications, and limitations of the findings.***

**Comment 8**

Conclusion: I disagree with your conclusion. It is very worrying that, despite the very limited data available and all the serious limitations, you end up concluding that “serious AEs” occur with solriamfetol. The side effects mentioned generally correspond to the known side effects and no real statement can be made about the frequency due to the given limitations. What are the serious AEs?

**Response: *Thank you for your critical observation. We have revised the conclusion to adopt a more cautious tone. We removed the term “serious adverse events” unless supported by specific outcomes (e.g., hospitalization, death) and acknowledged that most AEs reported are consistent with the known safety profile of solriamfetol from prior clinical trials. (P27 line 317-323)***

**Comment 9**

Most AEs are reported in the context of treatment for narcolepsy. This is not mentioned or discussed. Many patients with narcolepsy receive co- medication or polypharmacy. Another limitation, which is not mentioned.

**Response: *Thank you for your observation. We now emphasize that most AEs were reported in the context of narcolepsy treatment and that co-medication and polypharmacy are common in this population. This constitutes an important limitation, which we have added to the Discussion section. (P26 line 307-311)***

**Comment 10**

As further recommendation: In my opinion, it would be interesting if you could split the data sets into a part with results from consumer reports and a part with reports from physicians and compare results.

**Response: *Thank you for this thoughtful suggestion. We agree that comparing consumer-reported and physician-reported AEs could yield valuable insights. While our current dataset structure did not allow for a detailed stratified analysis, we will consider performing such a subgroup analysis in a future version of the study or in follow-up work.***

**Reviewer: 3**

**Comment 1**

The only point I am curious about is the details of serious adverse effects such as hospitalization and death. If possible, it would be very enlightening to add the details regarding these serious AEs, like why the patients were hospitalized or left disabled etc.
Other than that, I have no further comments. It was a pleasure to review this paper..

**Response: *Thank you for your insightful comment. We agree that providing further details on serious adverse events (SAEs) such as hospitalization and death would enrich the clinical relevance of our findings. However, due to the limitations of the FAERS database, detailed narrative information about the causes of hospitalization, death, or disability is often missing or nonspecific. In our dataset, many SAEs were associated with general or psychiatric adverse events (e.g., drug ineffectiveness, suicidal ideation, anxiety, and palpitations), but we were unable to consistently determine the direct reason for hospitalization or death.***

Thanks for your attention to our manuscript!

Sincerely,

Kaijian Zhu

Email: 13515245847@163.com
