# Supplementary material for: Post-marketing safety of solriamfetol: A retrospective pharmacovigilance study based on the us food and drug administration adverse event reporting system
Source: PLoS One. 2025 Sep 22;20(9):e0333130. doi: 10.1371/journal.pone.0333130 (PMC12453233; doi:10.1371/journal.pone.0333130)
Supplement: Attachment — Submitted filename: Response to Reviewers-2.docx [file pone.0333130.s006.docx]

**31-Jul-2025**

**Dear Prof. Christian Veauthier,**

Thank you for your time and valuable feedback on our manuscript entitled **"Post-marketing Safety of Solriamfetol: A Retrospective Pharmacovigilance Study Based on the US Food and Drug Administration Adverse Event Reporting System" (Submission ID PONE-D-25-14184R1)**. We have carefully addressed all comments and suggestions. Modifications in the revised manuscript are highlighted in red, and point-by-point responses are provided below:

**Additional Editor Comments:**
You have addressed the critical comments of the experts; unfortunately, methodological and substantive weaknesses continued to emerge during the review, which I would like to explain:
On the one hand, some reviewers criticized the fact that the data are not new and does not add any significant information to the data already published, but this is not a criterion for publication in Plos One.
Secondly, and more important, after inviting additional reviewers, there are also points of content that make publication in the current version impossible. The basic problem here seems to be that You are writing an article about a molecule (Solriamfetol) and not about a therapy for a distinct disorder. The situation would be completely different if the side effects of treating narcolepsy patients with Solriamfetol had been evaluated – or OSA patients with Solriamfetol…. But different indications are being lumped together here. Moreover, there seem to be also patients without narcolepsy or OSA? And there is little information about comorbidities. But the effects can only be meaningfully assessed if the indication and comorbidities are also known. Otherwise, this should have to be described as a serious methodological limitation (e.g. how many patients with elevated blood pressure already had elevated blood pressure before or were taking antihypertensives? Did this only occur in sleep apnea patients or also in narcolepsy patients?).

**Reviewer: 5**

**Comment 1**

As a clinician, I find it difficult to know exactly what to do with the data presented. Who is reporting in the FAERS and why? It is also not clear how this report on solriamfetol compares with reports on other similar drugs or drugs for similar indications.

For me, it is also not entirely clear from the current description exactly what kind of statistics were applied and how to fully understand them. But that is not crucial for the more general discussion of what to do with data from databases like this.

Apparently, most reports are from people who do not use solriamfetol for narcolepsy or OSAS. It then makes an addition, which is difficult for me to understand, that ‘the most frequently reported indication for solriamfetol was narcolepsy (596, 38.45%), followed by OSA (57, 3.68%)’. All other indications thus constitute < 3.68% of the total group. This implies that at least 15 other indications for solriamfetol were reported! Moreover, if I understand it correctly, the most often reported is that the drug is not effective. Personally, I would not interpret that as an AE.

Finally, methodological issues aside, what is reported on AEs is not really new or unexpected.

Nevertheless, the previous reviewer comments have been adequately addressed.

**Response to Editor and Reviewer #5:**

***We carefully analyzed the reviewers' and editors' comments and found that they were similar. We will reply together below:***

As a clinician, I find it difficult to know exactly what to do with the data presented. Who is reporting in the FAERS and why? It is also not clear how this report on solriamfetol compares with reports on other similar drugs or drugs for similar indications.

**Response: *We sincerely thank the reviewer for raising this important point. The primary objective of our study is to identify the most frequently reported AEs associated with solriamfetol based on data from the FAERS database. While we fully acknowledge that causality cannot be established from spontaneous reports, such analyses are widely used in pharmacovigilance to generate early safety signals and provide real-world insights that can inform clinical monitoring and future research.***

***Regarding the question of "who is reporting and why," we have clarified this in both the Methods section and Table 1, which provides reporter types. The majority of AE reports were submitted by healthcare professionals and consumers. We have now further emphasized this in the revised manuscript to improve clarity.***

***As for comparisons with other drugs used for similar indications (e.g., modafinil, pitolisant), we recognize the value of such analyses; however, our study was specifically designed to focus on solriamfetol and to explore its safety profile across all reported indications. Comparative analyses have been addressed in prior publications, and we consider them beyond the scope of the current investigation. We also have a brief summary in this article .***

For me, it is also not entirely clear from the current description exactly what kind of statistics were applied and how to fully understand them. But that is not crucial for the more general discussion of what to do with data from databases like this.

**Response: *We appreciate the reviewer’s comment regarding the statistical methods used. In this study, we employed four widely accepted disproportionality analysis algorithms: Reporting Odds Ratio (ROR), Proportional Reporting Ratio (PRR), Bayesian Confidence Propagation Neural Network (BCPNN), and Multi-item Gamma Poisson Shrinker (MGPS).***

***Each method offers distinct advantages:***

***– ROR is effective in correcting biases that may arise from low event counts.***

***– PRR provides higher specificity in detecting safety signals.***

***– BCPNN excels in integrating multi-source data and performing cross-validation.***

***– MGPS is particularly useful for identifying rare adverse events.***

***By combining these four methods, we aimed to enhance signal detection robustness and reliability. The joint use of multiple algorithms allows for cross-validation, helping to reduce false positives and increase the credibility of the findings. This strategy also broadens the detection scope and allows for the identification of potential rare adverse reactions through threshold adjustments and variance control.***

Apparently, most reports are from people who do not use solriamfetol for narcolepsy or OSAS. It then makes an addition, which is difficult for me to understand, that ‘the most frequently reported indication for solriamfetol was narcolepsy (596, 38.45%), followed by OSA (57, 3.68%)’. All other indications thus constitute < 3.68% of the total group. This implies that at least 15 other indications for solriamfetol were reported! Moreover, if I understand it correctly, the most often reported is that the drug is not effective. Personally, I would not interpret that as an AE.

**Response: *In response to the major concern regarding the heterogeneity of indications and lack of indication-specific analysis, we have added two new supplementary tables:***

- ***STable 2: Signal strength of adverse events associated with solriamfetol for the treatment of narcolepsy.***
- ***STable 3: Signal strength of adverse events associated with solriamfetol for the treatment of obstructive sleep apnea (OSA).***

***These tables allow for a clearer understanding of the safety profile of solriamfetol across its approved indications. The results show that while certain adverse events (e.g., drug ineffectiveness, headache, anxiety) are common to both indications, there are distinct signal differences in severity and ranking. We added a description of the corresponding content in the results section.***

***For reported indications. As shown in Table 1, we clearly listed the proportions of the two approved indications—narcolepsy (38.45%) and obstructive sleep apnea (3.68%)—in the included reports. However, 58.19% of the reports lacked specific indication information. This high percentage does not necessarily imply other indications. Rather, it reflects missing or incomplete data within the FAERS database, a common limitation in spontaneous reporting systems.***

***We agree that “drug ineffective” may not represent a physiological adverse event in the traditional sense. However, in pharmacovigilance practice, especially within spontaneous reporting systems like FAERS, “drug ineffective” is a commonly reported Preferred Term (PT) that reflects real-world dissatisfaction with therapeutic outcomes. While it does not necessarily indicate harm, a high frequency of such reports can serve as an important early signal for suboptimal drug response, inappropriate patient selection, dosing issues, or emerging patterns of therapeutic failure. Regulatory agencies such as the FDA consider these signals valuable for post-marketing surveillance and risk-benefit assessment.***

Finally, methodological issues aside, what is reported on AEs is not really new or unexpected.

**Response: *We thank the reviewer for this important observation. In response to this concern, we have added a new paragraph to the Discussion section comparing the adverse events (AEs) identified in our study with those listed in the official U.S. prescribing information for solriamfetol (Sunosi®). While several commonly reported AEs such as headache, anxiety, and insomnia are consistent with the product labeling, we also identified a number of previously underreported or unlabeled AEs—including therapeutic response decreased, irritability, suicidal ideation, mania, and persecutory delusion—which showed statistically significant signals in our FAERS analysis.***

***Importantly, through indication-specific stratified analysis, we found that psychiatric-related AEs were disproportionately reported in patients with narcolepsy, whereas cardiovascular-related AEs were more prominent in patients with OSA. These findings go beyond the current labeling and highlight indication-specific safety concerns that may not have been captured in pre-marketing clinical trials. We believe this adds novel, clinically relevant insights and underscores the value of post-marketing pharmacovigilance in complementing existing drug safety information.***

***We hope that these major revisions sufficiently address the methodological and interpretational concerns raised and bring the manuscript in line with the standards of PLOS ONE.***

Thanks for your attention to our manuscript!

Sincerely,

Kaijian Zhu

Email: 13515245847@163.com
