# Supplementary material for: Post-marketing safety of solriamfetol: A retrospective pharmacovigilance study based on the us food and drug administration adverse event reporting system
Source: PLoS One. 2025 Sep 22;20(9):e0333130. doi: 10.1371/journal.pone.0333130 (PMC12453233; doi:10.1371/journal.pone.0333130)
Supplement: Attachment — Submitted filename: Response to Reviewers-3.docx [file pone.0333130.s007.docx]

**22-Agu-2025**

**Dear Prof. Christian Veauthier,**

Thank you for your time and valuable feedback on our manuscript entitled **"Post-marketing Safety of Solriamfetol: A Retrospective Pharmacovigilance Study Based on the US Food and Drug Administration Adverse Event Reporting System" (Submission ID PONE-D-25-14184R2)**. We have carefully addressed all comments and suggestions. Modifications in the revised manuscript are highlighted in red, and point-by-point responses are provided below:

**Additional Editor Comments:**
The manuscript is significantly better and should be published soon, but there are still errors in Table 2:

The first column of Table 2 is labeled “Preferred Terms” and the second column is labeled “System Organ Class”. There seam to be obvious errors here: e.g., the organ “Renal and urinary disorders” is mentioned for the preferred term “cataplexy”. What does cataplexy have to do with renal and urinary disorders?

Or Sleep attacks with neoplasms? On the other hand, some information is incomprehensible: for example, why does “Suicidal intention” is mentionned under "investigations"? Readers cannot understand this without a comment in the legend.

The entire Table 2 appears to be full of errors. Please read carefully before resubmission all tables and ensure that the tables are error-free.

**Reviewer: 5**

**Comment 1**

The previous comments have been properly addressed.

But, something went wrong in Table S2: the second column is not correctly matched to the first column. Whether there is possibly also an error in the other columns I cannot determine.

**Response to Editor and Reviewer #5:**

***Thank you for your valuable feedback. We have carefully reviewed your comments and thoroughly re-examined the manuscript. Upon our careful recheck, we found that the issue was primarily confined to Table S2, where there was a misalignment between the "Preferred Terms" and the "System Organ Classes." This error was due to incorrect matching during the table preparation process, which led to the confusion, such as the association of "cataplexy" with "renal and urinary disorders" and "sleep attacks" with "neoplasms."***

***We have corrected the entries in Table S2 to ensure that the "Preferred Terms" are properly aligned with their corresponding "System Organ Classes." After reviewing the rest of the tables, we found no similar issues.***

***We believe these revisions have significantly improved the accuracy of the tables, and we have attached the revised manuscript with all necessary corrections. We hope these adjustments meet your expectations.***

Thanks for your attention to our manuscript!

Sincerely,

Kaijian Zhu

Email: 13515245847@163.com
