# Supplementary material for: Post-marketing safety of solriamfetol: A retrospective pharmacovigilance study based on the us food and drug administration adverse event reporting system
Source: PLoS One. 2025 Sep 22;20(9):e0333130. doi: 10.1371/journal.pone.0333130 (PMC12453233; doi:10.1371/journal.pone.0333130)
Supplement: Attachment — Submitted filename: Response to Reviewers-4.docx [file pone.0333130.s008.docx]

**7-Sep-2025**

**Dear Prof. Christian Veauthier,**

Thank you for your time and valuable feedback on our manuscript entitled **"Post-marketing Safety of Solriamfetol: A Retrospective Pharmacovigilance Study Based on the US Food and Drug Administration Adverse Event Reporting System" (Submission ID PONE-D-25-14184R3)**. We have carefully addressed all comments and suggestions. Modifications in the revised manuscript are highlighted in red, and point-by-point responses are provided below:

**Editor Comments:**
Unfortunately, I have one more comment to make:

in Table 2, the preferred term “sleepiness” is assigned to system class “psychiatric disorder.”

I find this difficult to understand given that the article is about narcolepsy and daytime sleepiness,

and it reveals that the authors have no concept of daytime sleepiness and think that sleepiness is psychologically induced. That is not correct: Sleep apnea can cause sleepiness. Narcolepsy can cause sleepiness as well: neither is a psychiatric disorder. Sleepiness per se is not psychiatric. Please change the organ class.

**Response: *We sincerely thank the editor for pointing out this important issue. We fully agree that sleepiness is not a psychiatric disorder, and conditions such as narcolepsy or obstructive sleep apnea can lead to excessive sleepiness through non-psychiatric mechanisms. I will now provide a detailed explanation.***

***We carefully reviewed our data and would like to clarify that in our Table 2, only the SOCs are presented, and the Preferred Term “sleepiness” does not actually appear in our tables. All adverse events were coded strictly according to the standardized MedDRA dictionary, which automatically maps PT to predefined SOCs. In MedDRA, some terms related to excessive daytime sleepiness (e.g., sleep attacks) may be automatically classified under the SOC “psychiatric disorders,” even though clinically these events are primarily neurological or sleep–wake phenomena rather than psychiatric in nature. This point has already been introduced in the Methods section. Therefore, the classification reflects the MedDRA structure rather than our interpretation.***

***In our analysis, we first presented the results at the SOC level to give a global overview of the organ systems most frequently involved. However, as the editor correctly pointed out, the SOC classification is based on MedDRA conventions and may sometimes group sleep-related events (e.g., sleep attacks) under “psychiatric disorders,” even though clinically they are not psychiatric in nature. To address this limitation and provide readers with a more accurate understanding, we also performed a PT–level analysis and explicitly listed the PTs together with their assigned SOCs in MedDRA structure. By doing so, readers can clearly see the specific events that contributed to each SOC signal and avoid misinterpreting sleep-related symptoms as psychiatric disorders.***

***In the revised manuscript, we have added clarifications in the Discussion to emphasize that SOC assignment follows MedDRA rules, while the PT-level analysis allows a more nuanced interpretation in line with clinical reality. We believe this approach enhances transparency and directly addresses the editor’s concern.***

***We sincerely thank the editor again for highlighting this important issue, which has helped us improve the clarity and rigor of our manuscript.***

Thanks for your attention to our manuscript!

Sincerely,

Kaijian Zhu

Email: 13515245847@163.com
